# Supplementary material for: Active removal of inhibitory components drives the flagellar Type III Secretion Specificity Switch
Source: bioRxiv. 2026 Mar 10:2026.03.09.710671. Preprint. [Version 1] doi: 10.64898/2026.03.09.710671 (PMC13060781; doi:10.64898/2026.03.09.710671)
Supplement: Supplement 1 [file media-1.pdf]

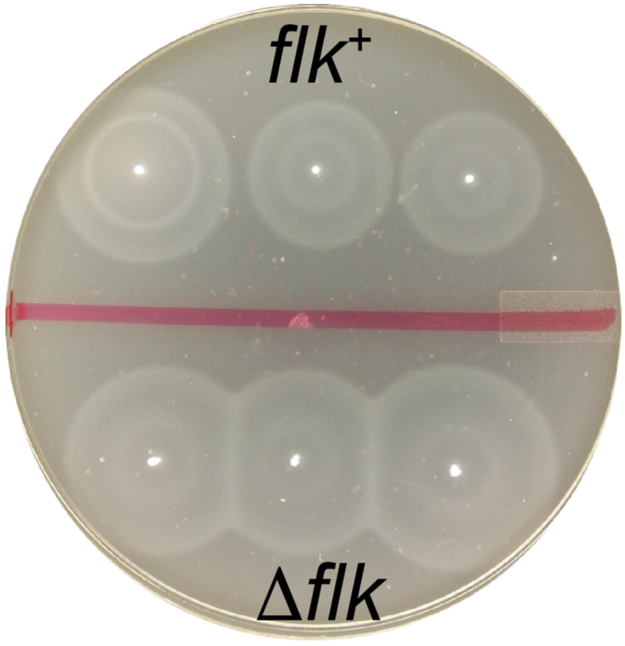

Supplemental Figure 1. The deletion of *flk* has no apparent effect on *Salmonella* motility (*flk*$^+$ = strain TH437 (LT2); Δ*flk* = strain TH1460).

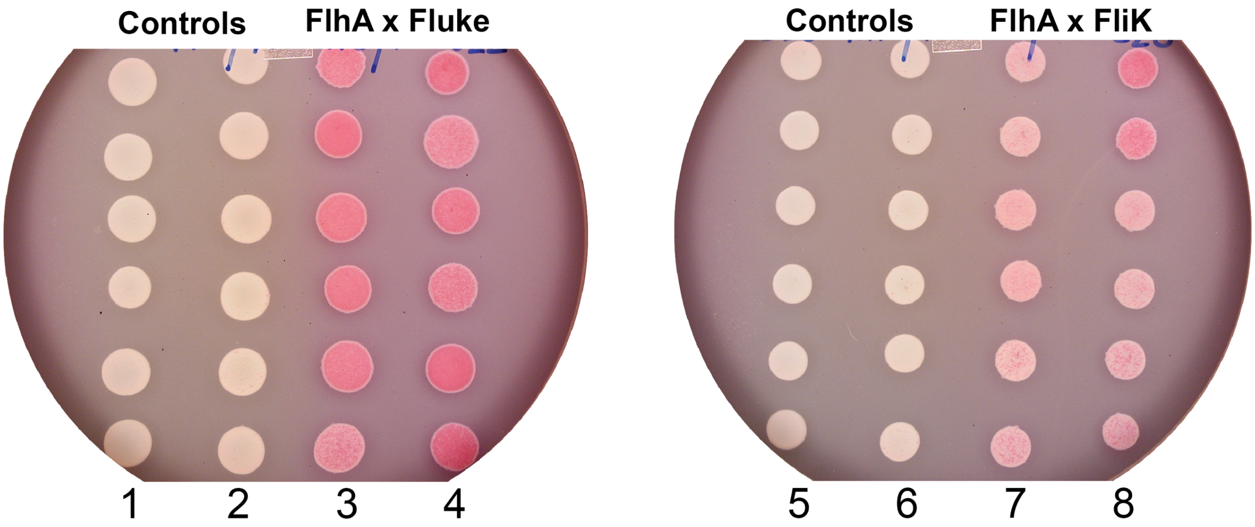

Supplemental Figure 2. A bacterial two hybrid screen for interaction between either Fluke or FliK with $FlhA_C$ indicates a positive interaction phenotype (Lac^+ pink color formation). Lane 1= pUT18C-FliK x pKNT25 empty vector; lane 2= pUT18C empty vector x pKNT25-FlhA C-terminus; lanes 3 and 4 = pUT18C-Fluke x pKNT25-FlhA C-terminus; lane 5= pUT18C-Fluke x pKNT25 empty vector; lane 6= pUT18C empty vector x pKNT25-FlhA C-terminus; lanes 7 and 8 = pUT18C-FliK x pKNT25-FlhA C-terminus.

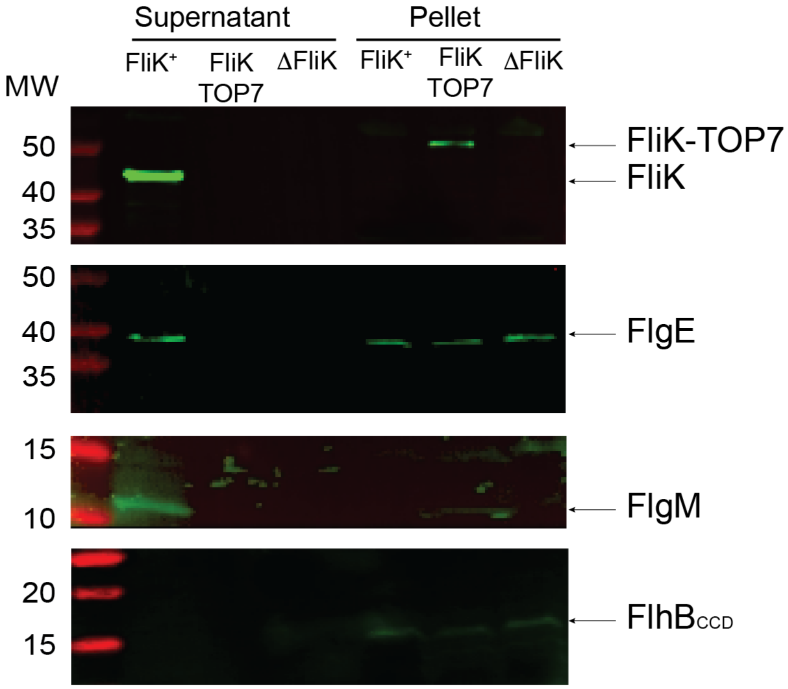

Supplemental Figure 3. The cleavable C-terminal domain of FlhB is not detected in the cell supernatant, just after the secretion specificity switch has occurred. Cells (1L) were grown to OD 0.5. Cells were pelleted, washed once and resuspended into 20 ml of media containing arabinose and induced for 15 mins. Supernatant was concentrated to 200 µl and 20 µl/lane was loaded.

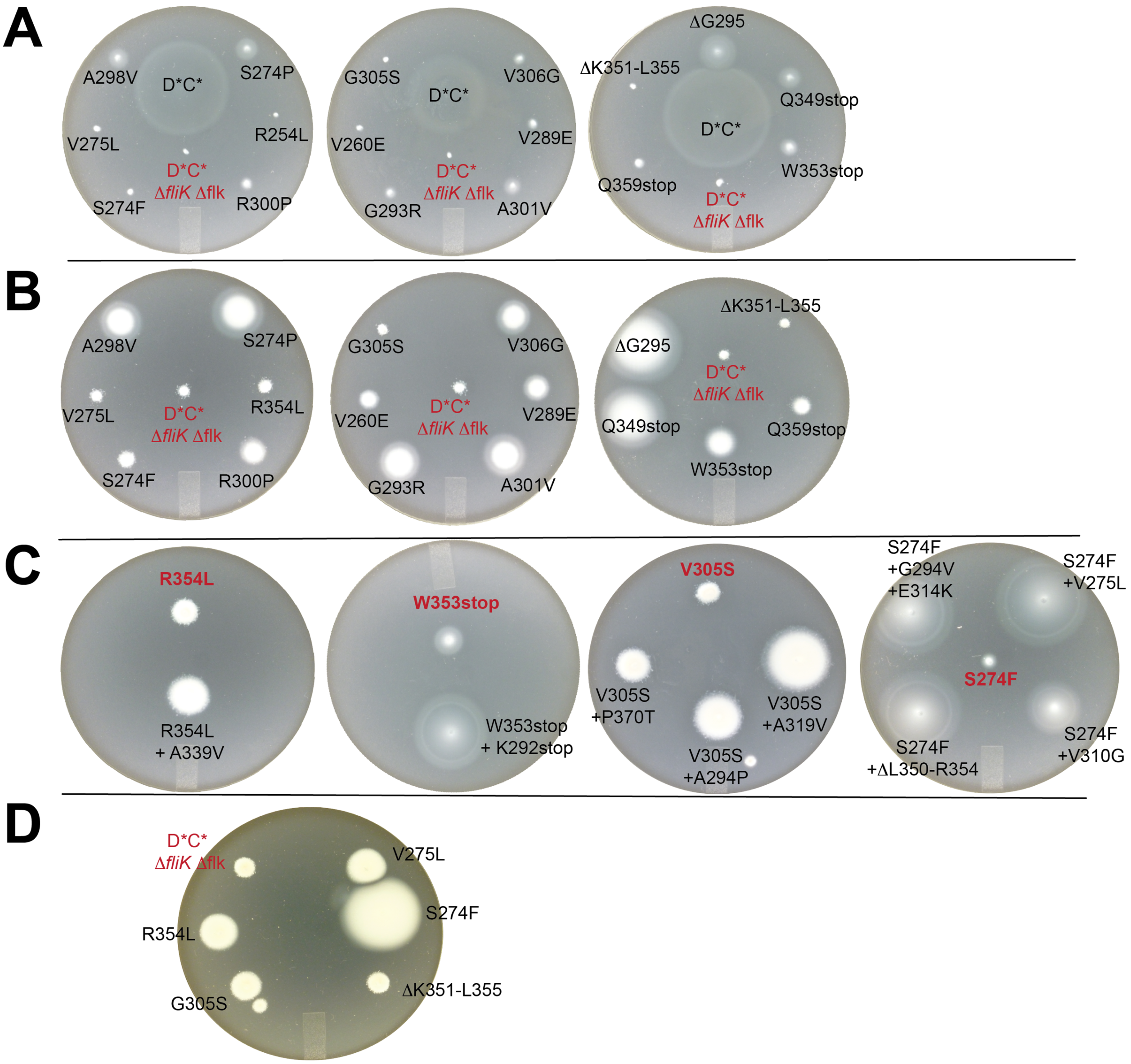

Supplemental Figure 4. Motile revertants of Δ*fliK* Δ*flk* double mutant strain in the *flhD*C background TH29848 (*flhD8070*(L22H) *flhC8092*(Q29P) Δ*fliK9249* Δ*flk-7755*). A.** Motility relative to the parent TH29848 (labeled red) and a *fliK*$^{+}$ *flk*$^{+}$ motile strain showing that motile revertants do not exhibit full "wild-type" motility after 4 hours incubation in soft agar motility plates at 37°C. **B.** Motility phenotypes relative to the parent strain (red), TH29848, after 8 hours incubation in soft agar medium at 37°C. **C.** Double-mutants with increased motility shown relative to the original motile revertant phenotype (red) after 10 hours incubation in soft agar motility plates at 37°C. **D.** These are motility revertants with only slight increased motility phenotypes after 16 hours incubation on soft agar motility plates at 37°C.

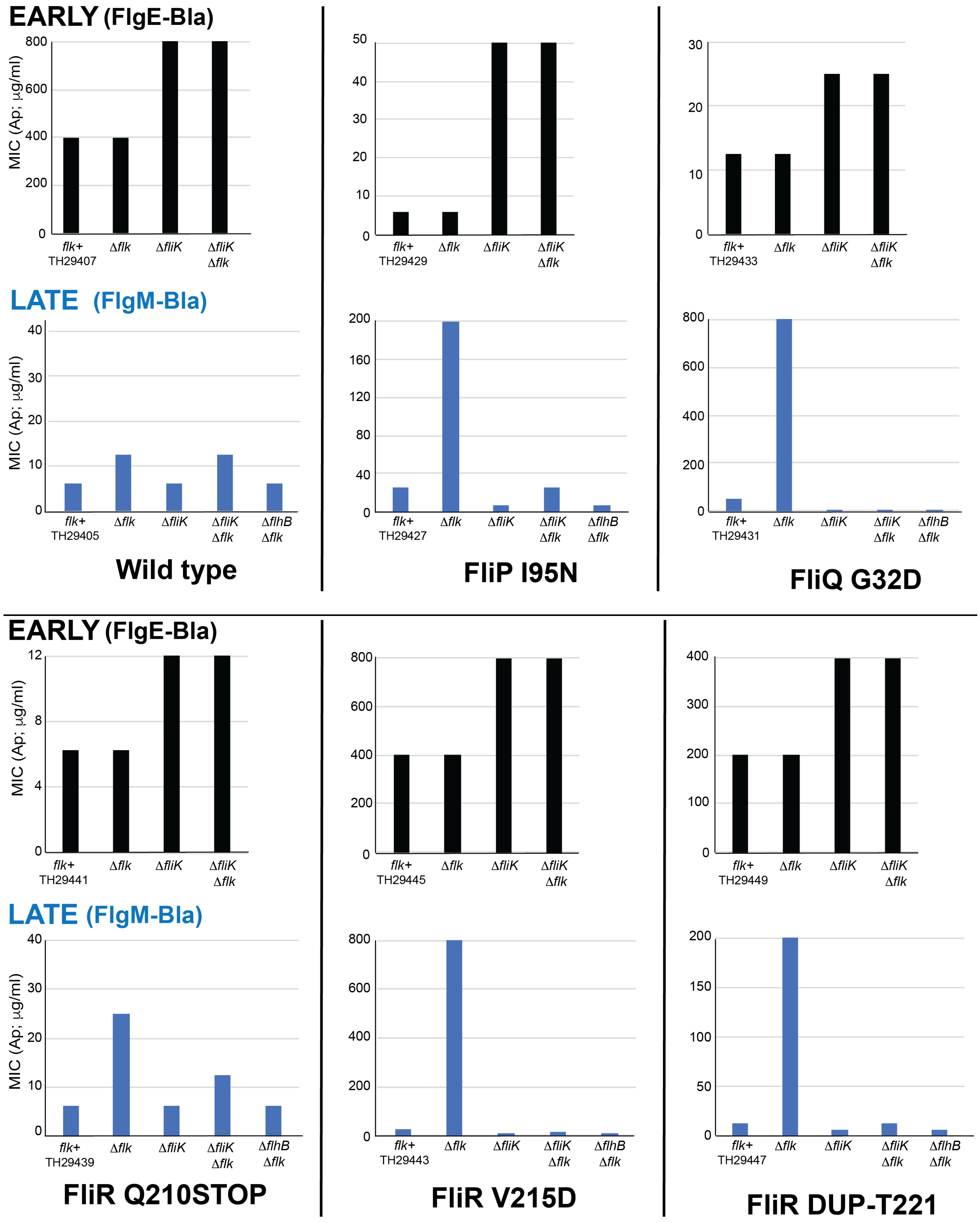

Supplemental Figure 5. Levels of early (FlgE-Bla) and late (FlgM-Bla) substrate secretion in the FlhB-bypass mutants in the presence and absence of Fluke, and/or FliK. Parent strain numbers (*flk*$^{+}$) are indicated in the figure. Genotypes of all strains used are listed in Table S4.

Supplemental Tables

Table S1. Plasmids and Bacterial strains used in this study*

Plasmids:

Plasmid Name	Usage and relevant characteristic	Source*
pKD3	For inserting FRT-Cm-FRT chromosomal cassette, Ap^R plasmid	(13)
p-Sim5	Temperature inducible λ-Red expressing plasmid, Cm^R plasmid, pSC101 origin of replication (replicates at 30°C)	(14)
p-Sim-6	Temperature inducible λ-Red expressing plasmid, Ap^R plasmid, pSC101 origin of replication (replicates at 30°C)	(14)
p-EM5	Km^R P_{nahG}-*fliQ*$^+$	M. Erhardt

E.coli **strains:**

***E.coli* Strains**	Genotype	Source*
MG1655 MGRFC	Δ*leuB motA*(E98K) Int5::Zeo^R-PRNA-mcherry *galK*::P_{fliF}(*E. coli*)-CFP-Ap^R *attB*::P_{fliC}(*E. coli*)-YFP-Km^R	(33)
TH408	*nad*::Tn*10d*Tc	Lab collection
BTH101	F^- *cya-99 araD139 galE15 galK16 rpsL1*(Str^R) *hsdR2 mcrA1 mcrB1*	BACTH, Euromedex

Salmonella **Typhimurium strains:**

Salmonella strains	Genotype	Source*
TH437	LT2 *Salmonella* wild type strain	J.Roth
TH9949	*flg*E*6569*::*bla* Δ*flgBC6557*	Lab collection
TH13359	*fliK7582*::*bla*	Lab collection
TH13807	*fliK7582*::*bla* Δ*flgA7656* (Δamino acids 6-214 (of 219))	Lab collection
TH13808	*fliK7582*::*bla* Δ*flgB7657* (Δamino acids 6-133(of 138))	Lab collection
TH13809	*fliK7582*::*bla* Δ*flgC7658* (Δamino acids 6-129(of 134))	Lab collection
TH13810	*fliK7582*::*bla* Δ*flgD6540* (Δamino acids 2-220(of 232))	Lab collection
TH13811	*fliK7582*::*bla* Δ*flgE7659* (Δamino acids 6-398(of 403))	Lab collection
TH13812	*fliK7582*::*bla* Δ*flgF7660* (Δamino acids 6-246(of 251))	Lab collection
TH13813	*fliK7582*::*bla* Δ*flgG7661* (Δamino acids 6-255(of 260))	Lab collection
TH13814	*fliK7582*::*bla* Δ*flgH7662* (Δamino acids 6-227(of 232))	Lab collection
TH13815	*fliK7582*::*bla* Δ*flgI7663* (Δamino acids 6-360(of 365))	Lab collection
TH13816	*fliK7582*::*bla* Δ*flgJ7664* (Δamino acids 6-311(of 316))	Lab collection
TH13817	*fliK7582::bla* Δ*flgK7665* (Δamino acids 6-548(of 553))	Lab collection
TH13818	*fliK7582*::*bla* Δ*flgL7666* (Δamino acids 6-312(of 317))	Lab collection
TH14607	Δ*flk-7755*	Lab collection
TH23959	Δ*flhB8612*$_{\Delta CCD}$ (*flhB*$_{\Delta CCD}$ = deletion of amino acids P270-G383)	Lab collection
TH24321	Δ*flgBC6557 flgE6569*::*bla* Δ*flk-7755*	
TH24658	Δ*flgBC6557 flgE6569*::*bla* Δ*flk-7755*	
TH24722	*flgM6427::bla* Δ*flgB-L8735* Δ*flhB8612*$_{\Delta CCD}$ *flhD8070 flhC8092 fliA5225*(H14D) Δ*fliB-T7771 fljB*^{enx} *vh2*	
TH24787	*flgM6427::bla* Δ*flgB-L8735* Δ*flhB8612*$_{\Delta CCD}$ *flhD8070 flhC8092 fliA5225*(H14D) Δ*fliB-T7771* Δ*flk-7755 fljB*^{enx} *vh2*	
Salmonella strains	Genotype	Source*
TH24789	Δ*araBAD961*::*flk*$^{+}$ *flgM6427::bla* Δ*flgB-L8735* Δ*flhB8612*$_{\Delta CCD}$ *flhD8070 flhC8092 fliA5225*(H14D) Δ*fliB-T7771 fljB*^{enx} *vh2*	
TH24800	*flgM6427*::*bla* Δ*flgB-L8735* Δ*flhB8612*$_{\Delta CCD}$ *flhD8070 flhC8092 fliA5225*(H14D) Δ*fliB-T7771* Δ*fliK6140* Δ*flk-7755 fljB*^{enx} *vh2*	
TH24819	*flgM6427*::*bla* Δ*flgB-L8735* Δ*flhB8612*$_{\Delta CCD}$ *flhD8070 flhC8092 fliA5225*(H14D) Δ*fliB-T7771* Δ*fliK6140 fljB*^{enx} *vh2*	

TH25226	*flgM6427*::*bla* Δ*flgB-L8735 flhD8070 flhC8092 fliA5225*(H14D) Δ*fliB-T7771* Δ*flk-7755 fljB*^{enx} *vh2*	
TH25935	*flgM6427*::*bla* ΔflgB-L8735 *flhB8612*$_{\Delta CCD}$ *fljB*^{enx} *vh2*	
TH25936	Δ*flgBC6557 flgE6569*::*bla flhB861*$_{\Delta CCD}$ *2 fljB*^{enx} *vh2*	
TH25938	*flgM6427*::*bla* Δ*flgB-L8735 flhB8612*$_{\Delta CCD}$ Δ*flk-7755 fljB*^{enx} *vh2*	
TH25939	Δ*flgBC6557 flgE6569*::*bla flhB8612*$_{\Delta CCD}$ Δ*flk-7755 fljB*^{enx} vh2	
TH26028	*flgM6427*::*bla* Δ*flgB-L8735 flhB8612*$_{\Delta CCD}$ *fliK6620* Δ*flk-7755 fljB*^{enx} *vh2*	
TH26992	*flgM6427*::*bla* Δ*flgB-L8735 STM1911*::Tn*10d*Tc Δ*flhBAE7670*::FCF *flhD8070 flhC8092 fliA5225*(H14D) Δ*fliB-T7771* Δ*flk-7755 fljB*^{enx} *vh2*	
TH27083	pSIM5/LT2	
TH27122	Δ*araBAD957*::*rflP*$^{+}$ Δ*rflM8403* P$_{flhDC}$*5451*::TPOP *fljB*^{enx} *vh2*	
TH27152	*flgM6427*::*bla* Δ*flgB-L8735 flhD8070 flhC8092 fliA5225*(H14D) Δ*fliB-T7771* Δ*fliQ8223*::*tetRA* (Δ codons 31-50) Δflk-7755 *fljB*^{enx} *vh2*	
TH27337	*attB*::Cm^{R}-P$_{fliF}$-YFP *argW*::Zeo^{R}-mcherry Δ*galK*::Ap^{R}-P$_{fliC}$-CFP P$_{flhDC}$*5451*::TPOP *fljB*^{enx} *vh2*	
TH27343	*attB*::Cm^{R}-P$_{fliF}$-YFP *argW*::Zeo^{R}-mcherry Δ*galK*::Ap^{R}-P$_{fliC}$-CFP *flgM7929*::TPOP *fljB*^{enx} *vh2*	
TH27622	P$_{flhDC}$*5451*::TPOP Δ*rflM8403 fljB*^{enx} *vh2*	
TH27623	P$_{flhDC}$*5451*::TPOP Δ*rflM8403* Δ*fliK6140 fljB*^{enx} *vh2*	
TH27965	Δ*araBAD2101*::*flhB*$^{+}$ *flgM6427*::*bla* Δ*flgB-L8735 flhD8070 flhC8092 fliA5225*(H14D) Δ*fliB-T7771* Δ*flk-7755 fljB*^{enx} *vh2*	
TH28049	*attB*::Cm^{R}-P$_{fliF}$-YFP Δ*galK*::Ap^{R}-P$_{fliC}$-CFP *fljB*^{enx} *vh2*	
TH28050	*attB*::Cm^{R}-P$_{fliF}$-YFP Δ*galK*::Ap^{R}-P$_{fliC}$-CFP Δ*flgHI958 fljB*^{enx} *vh2*	
TH28051	*attB*::Cm^{R}-P$_{fliF}$-YFP Δ*galK*::Ap^{R}-P$_{fliC}$-CFP *flhB8612 fljB*^{enx} *vh2*	
TH28052	*attB*::Cm^{R}-P$_{fliF}$-YFP Δ*galK*::Ap^{R}-P$_{fliC}$-CFP Δ*flk-7755 fljB*^{enx} *vh2*	
TH28053	*attB*::Cm^{R}-P$_{fliF}$-YFP Δ*galK*::Ap^{R}-P$_{fliC}$-CFP Δ*flgHI958* Δ*flk-7755 fljB*^{enx} *vh2*	
TH28054	*attB*::Cm^{R}-P$_{fliF}$-YFP Δ*galK*::Ap^{R}-P$_{fliC}$-CFP *flhB8612*$_{\Delta CCD}$ Δ*flk-7755 fljB*^{enx} *vh2*	
TH28055	*attB*::Cm^{R}-P$_{fliF}$-YFP Δ*galK*::Ap^{R}-P$_{fliC}$-CFP *flhB8612*$_{\Delta CCD}$ Δ*flgHI958 fljB*^{enx} *vh2*	
TH28056	*attB*::Cm^{R}-P$_{fliF}$-YFP Δ*galK*::Ap^{R}-P$_{fliC}$-CFP *flhB8612*$_{\Delta CCD}$ Δ*flgHI958* Δ*flk-7755 fljB*^{enx} *vh2*	
TH28157	*flgM6427*::*bla* Δ*flgB-L8735 fljB*^{enx} *vh2*	
TH28158	*flgM6427*::*bla* Δ*flgB-L8735* Δ*flk-7755 fljB*^{enx} *vh2*	
TH29063	Δ*araBAD2101*::*flhB*$^{+}$ *flgM6427*::*bla* Δ*flgB-L8735 flhB8612*$_{\Delta CCD}$ Δ*flk-7755 fljB*^{enx} *vh2*	
TH29064	Δ*araBAD2101*::*flhB*$^{+}$ Δ*flgBC6557 flgE6569*::*bla flhB8612*$_{\Delta CCD}$ Δ*flk-7755 fljB*^{enx} *vh2*	
TH29116	Δ*flgBC6557 flgE6569*::*bla* Δ*flhB8612*$_{\Delta CCD}$ Δ*fliK9249* Δ*flk-7755*	
TH29314	*attB*::Cm^{R}-P$_{fliF}$-YFP Δ*galK*::Ap^{R}-P$_{fliC}$-CFP Δ*flk-7755 fljB*^{enx} *vh2*	
TH29315	*attB*::Cm^{R}-P$_{fliF}$-YFP Δ*galK*::Ap^{R}-P$_{fliC}$-CFP *flhB7152*(N269A) Δ*flgHI958 fljB*^{enx} *vh2*	
TH29316	*attB*::Cm^{R}-P$_{fliF}$-YFP Δ*galK*::Ap^{R}-P$_{fliC}$-CFP *flhB7152*(N269A) Δ*flgHI958* Δ*flk-7755 fljB*^{enx} *vh2*	
TH29317	*attB*::Cm^{R}-P$_{fliF}$-YFP Δ*galK*::Ap^{R}-P$_{fliC}$-CFP *flhB8612*$_{\Delta CCD}$ *flhD8070 flhC8092* Δ*flk-7755 fljB*^{enx} *vh2*	
TH29318	*attB*::Cm^{R}-P$_{fliF}$-YFP Δ*galK*::Ap^{R}-P$_{fliC}$-CFP *flhB8612*$_{\Delta CCD}$ *flhD8070 flhC8092* Δ*flgHI958 fljB*^{enx} *vh2*	
Salmonella strains	Genotype	Source*

TH29319	*attB*::Cm^{R}-P$_{fliF}$-YFP Δ*galK*::Ap^{R}-P$_{fliC}$-CFP *flhB8612*$_{\Delta CCD}$ *flhD8070 flhC8092* Δ*flgHI958* Δ*flk-7755 fljB*^{enx} *vh2*	
TH29357	*attB*::Cm^{R}-P$_{fliF}$-YFP Δ*galK*::Ap^{R}-P$_{fliC}$-CFP Δ*flhB8789 (*leaves first 5 and last 9 amino acids of FlhB) *flhD8070 flhC8092* Δ*flk-7755 fljB*^{enx} *vh2*	
TH29358	*attB*::Cm^{R}-P$_{fliF}$-YFP Δ*galK*::Ap^{R}-P$_{fliC}$-CFP Δ*flgHI958 flhB8789 flhD8070 flhC8092 fljB*^{enx} *vh2*	
TH29359	*attB*::Cm^{R}-P$_{fliF}$-YFP Δ*galK*::Ap^{R}-P$_{fliC}$-CFP Δ*flgHI958 flhB8789 flhD8070 flhC8092* Δ*flk-7755 fljB*^{enx} *vh2*	
TH29848	*flhD8070 flhC8092* Δ*fliK9249* Δ*flk-7755*	
TH30093	Δ*ara*BAD7606::*fliK*$^{+}$ Δ*fliK9249 attB*::Cm^{R}-P$_{fliF}$-YFP Δ*galK*::Ap^{R}-P$_{fliC}$-CFP *flhD8070 flhC8092* Δ*prgH73*::*tetRA ompT*::Km *fljB*^{enx} *vh2*	
TH30110	Δ*araBAD7609*::*fliK*-TOP7 Δ*fli*K9249 *attB*::Cm^{R}-P$_{fliF}$-YFP Δ*galK*::Ap^{R}-P$_{fliC}$-CFP *flhD8070 flhC8092* Δ*prgH73*::*tetRA ompT*::Km *fljB*^{enx} *vh2*	
TH30111	Δ*araBAD937*::FKF Δ*fliK9249 attB*::Cm^{R}-P$_{fliF}$-YFP Δ*galK*::Ap^{R}-P$_{fliC}$-CFP *flhD8070 flhC8092* Δ*prgH73*::*tetRA ompT*::Km *fljB*^{enx} *vh2*	
TH30250	Δ*flgBC6557 flgE6569*::*bla* Δ*flhB8612*$_{\Delta CCD}$ Δ*fliK9249*	
TH30367	*fliK9504*(Δaa311-320) *fliK7582*::*bla*	
TH30368	*fliK9505*(P296L) *fliK7582*::*bla*	
TH30413	Δ*galK*::P$_{fliC}$-*luxCDBAE*-Cm^{R} P$_{flhDC}$*5451*::Tn*10d*Tc[*del-25*] *fliK7582*::*bla*	
TH30414	Δ*galK*::P$_{fliC}$-*luxCDBAE*-Cm^{R} P$_{flhDC}$*5451*::Tn*10d*Tc[*del-25*] *fliK9504*(Δaa311-320) *fliK7582*::*bla*	
TH30415	Δ*galK*::P$_{fliC}$-*luxCDBAE*-Cm^{R} P$_{flhDC}$*5451*::Tn*10d*Tc[*del-25*] *fliK9505*(P296L) *fliK7582*::*bla*	

*Unless indicated otherwise these strains were constructed during this work

Salmonella enterica serovar Typhimurium **14028 strains:**

Salmonella strains	Genotype	Source*
TH27356	14028 *argW*::Zeo^{R}-PmCherry *attB*::Km^{R}-P$_{fliF}$-YFP Δ*galK*::Ap^{R}-P$_{fliC}$-CFP Δ*prgH74* Δ*ssaN109* Δ*flgHI958 motA8739*(E98K) Δ*fliC7716 fljB*^{enx} *vh2*	
TH27359	14028 *argW*::Zeo^{R}-PmCherry *attB*::Km^{R}-P$_{fliF}$-YFP Δ*galK*::Ap^{R}-P$_{fliC}$-CFP Δ*prgH74* Δ*ssaN109* Δ*flgHI958* Δ*flk*-7755 *motA8739*(E98K) Δ*fliC7716 fljB*^{enx} *vh2*	
TH27476	14028 *argW*::Zeo^{R}-PmCherry *attB*::Km^{R}-P$_{fliF}$-YFP Δ*galK*::Ap^{R}-P$_{fliC}$-CFP Δ*prgH74* Δ*ssaN109* Δ*flgHI958* Δ*flk-7755 flhB8612*$_{\Delta CCD}$ *motA8739* Δ*fliC7716 fljB*^{enx} *vh2*	
TH27477	14028 *argW*::Zeo^{R}-PmCherry *attB*::Km^{R}-P$_{fliF}$-YFP Δ*galK*::Ap^{R}-P$_{fliC}$-CFP Δ*prgH74* Δ*ssaN109* Δ*flgHI958 flhB8612 motA8739* Δ*fliC7716 fljB*^{enx} *vh2*	
TH27478	14028 *argW*::Zeo^{R}-PmCherry *attB*::Km^{R}-P$_{fliF}$-YFP Δ*galK*::Ap^{R}-P$_{fliC}$-CFP Δ*prgH74* Δ*ssaN109* Δ*flgHI958* Δ*flk-7755* Δ*fliK6620 motA8739* Δ*fliC7716 fljB*^{enx} *vh2*	
TH27451	14028 *argW*::Zeo^{R}-PmCherry *attB*::Km^{R}-P$_{fliF}$-YFP Δ*galK*::Ap^{R}-P$_{fliC}$-CFP Δ*prgH74* Δ*ssaN109* Δ*flgM5628*::FRT Δ*flgHI958 motA8739* Δ*fliC7716 fljB*^{enx} *vh2*	

Table S2. Oligonucleotides used in this study

Name	Sequence (from 5'- to 3'-)
1874-flhBCLtetR	gatggaagatgtgccgaaagcggacgtcattgtcactaacttaagacccactttcacatt
1910-flhB375tetA	cgcgaccagattagccatcagtattcttctcgttcataaactaagcacttgtctcctg
1911-flhBccDel-A	gcgcagcgccgcatgatggaagatgtgccgaaagcggacgtcattgtcactaactaattt
1912-flhBccDel-B	cgcagcatcgcgaccagattagccatcagtattcttctcgttcataaattagttagtgac
4543-fliKfullbla_rv	gatcttcagcatcttttactttcaccagcgtttctgggtgggcgaagatatccactgcgc
7516-FliK278	gtcatgttatttacgcgtcag
8283-galK-sCFP3A	cgcggtcagcgacatccattttcgcgaatccggagtaTAAaaAGGTCTAGGCGGCGCCTA
8284-galK-amp-cfp3A	cctgctccttgtgacggtttgcatacataaaaggtttcTTACCAATGCTTAATCAGTGAGGC
8285-ybhCvenusNB	gcctgaaaaggaactttttaccttttcgccttcccgtttcgtGGCAGCAAAACCCGTACC
8286-ybhC venusNBKan	agttaaatgacatccattgaagcctgctttttttatactaagttgaGCTTGGATTCTCACC
8309-attB-Km_Prom-venusNB-tetR	acctataaaaataggcgtatcacgaggccctttcgtcttgac TTAAGACCCACTTTCACATT
8315-galKtetR-scCFP3A	atttgaatgtatttagaaaaataaacaaataggggttccgcgTTAAGACCCACTTTCACATT
8331-galK-tetA-CFP	ggtctagactccttactaaagttaaacaaaattattatcaatCTAAGCACTTGTCTCCTG
8458-galK-PFliCsal	atttgaatgtatttagaaaaataaacaaataggggttccgcgGTTCTTTGTCAGGTCTGTC
8459-attB-Km_PfliF-salm-venusfw	actcatatgtatatctccttcttaaagttaaacaaaattatt GGATTCGCGCGTAGGCGA
8460-attB-Km_PfliFsal-venusrv	cctataaaaataggcgtatcacgaggccctttcgtcttgac CGTCGACTGCGAGTGC
9083-new-attB-km-promvenusNBtetA	actcatatgtatatctccttcttaaagttaaacaaaattattCTAAGCACTTGTCTCCTG
9142-galK-PFliC-RBSforCFP	ggtctagactccttactaaagttaaacaaaattattatcaatCGCAGACCGGAAGACAGA
9305-attB-Cm-rv	tgagcttggattctcaccaataaaaaacgcccggcggcaaccCACTCATCGCAGTACTGTTGTAT
9306-attB-Cm-fw	tttcttagacgtcggaattgccagctggggcgccctctggTCCTGGTGTCCCTGTTGATac
9318-FliQtetR	tctcgccctggctgcgccgctgttactcgtcgcgctgattttaagacccactttcacatt
9319-FliQtetA	aacgataattgcgatgaataccgcgacgattttagggataaactaagcacttgtctcctg
9320-FliQ-AA31-50dopped	gttactcgtcgcgctgattaccggcctcattatcagcatcttgcaggccgcgactcagattaatgaaatgacgctgtcgtttatc cctaaaatcgtcgcg
9321-fliQ50-fill	cgcgacgattttagggat
9322-FliQ-30fw	agtcgctctcgccctggctgcgccgctgttactcgtcgcgctgat
9323-FliQ-50-rev	ggcaacgataattgcgatgaataccgcgacgattttagggat

Table S3. Class 3 promoter activity relative to class 2 promoter activity in individual bacterial cells, in different genetic backgrounds

Strain Number	Relevant Genotype (all strains contained the P_{fliF}-*yfp* P_{fliC}-*cfp*)	Number of cells expressing YFP*	Number of cells expressing CFP*	Percentage Class3/2
TH28049	wild type	992	989	100
TH27343	*flgM* null	853	854	100
TH28052	Δ*flk*	874	866	99
TH28050	Δ*flgHI*	1016	1	<0.01
TH28051	$flhB_{\Delta CCD}$	1015	18	1.8
TH28054	Δ*flk* $flhB_{\Delta CCD}$	654	12	1.8
TH29314	Δ*flk* *flhB*N269A	1305	20	1.5
TH28053	Δ*flgHI* Δ*flk*	1405	288	20.5
TH28055	Δ*flgHI* $flhB_{\Delta CCD}$	954	3	0.3
TH29315	Δ*flgHI* *flhB*N269A	1560	0	<0.01
TH28056	Δ*flgHI* Δ*flk* $flhB_{\Delta CCD}$	704	14	2
TH29316	Δ*flgHI* Δ*flk* *flhB*N269A	998	2	0.2
TH29317	*flhD*C** $flhB_{\Delta CCD}$ Δ*flk*	946	764	90
TH29318	*flhD*C** $flhB_{\Delta CCD}$ Δ*flgHI*	1180	1	0.1
TH29319	*flhD*C** $flhB_{\Delta CCD}$ Δ*flk* Δ*flgHI*	1480	1465	99
TH29357	*flhD*C** Δ*flhB* Δ*flk*	950	0	<0.01
TH29358	*flhD*C** Δ*flhB* Δ*flgHI*	1000	0	<0.01
TH29359	*flhD*C** Δ*flhB* Δ*flk* Δ*flgHI*	1200	0	<0.01

*Cells were imaged at cell density of approximately 1.

Table S4. Strains used in Figure 7 and supplemental Figure 5:

<u>FlgM-Bla</u> strains:
All strains contain *flgM6407*::*bla* Δ*flgB-L8735 fliA5225* Δ*fliB-T7771 fljB*^{enx} *vh2*

	WT	FliP (I95N)	FliQ (G32D)	FliR (Q210 stop)	FliR (V215D)	FliR (T221DUP)
flk^{+}	TH29405	TH29427	TH29431	TH29439	TH29443	TH29447
Δ*flk*	TH29406	TH29428	TH29432	TH29440	TH29444	TH29448
Δ*fliK*	TH29684	TH29451	TH29455	TH29463	TH29467	TH29471
Δ*fliK* Δ*flk*	TH29685	TH29452	TH29456	TH29464	TH29468	TH29472

<u>FlgE-Bla</u> strains:
All strains contain *flgE6569*::*bla* Δ*flgBC6557 fliA5225* Δ*fliB-T7771 fljB*^{enx} *vh2*

	WT	FliP (I95N)	FliQ (G32D)	FliR (Q210 stop)	FliR (V215D)	FliR (T221DUP)
flk^{+}	TH29407	TH29429	TH29433	TH29441	TH29445	TH29449
Δ*flk*	TH29408	TH29430	TH29434	TH29442	TH29446	TH29450
Δ*fliK*	TH29686	TH29453	TH29457	TH29465	TH29469	TH29473
Δ*fliK* Δ*flk*	TH29687	TH29454	TH29458	TH29466	TH29470	TH29474

<u>FliK-Bla</u> strains:
All strains contain *fliK7582*::*bla flhB7152*(N269A) Δ*flgB-L8735 fliA5225* Δ*fliB-T7771 flhD8070 flhC8092 fljB*^{enx} *vh2*

	WT	FliP I95N	FliQ (G32D)	FliR Q210 stop	FliR T221DUP	FliR V215D	FliK P296L	FliK Δ311-320
flk^{+}	TH30353	TH30354	TH30355	TH30357	TH30359	TH30358	TH30405	TH30403
Δ*flk*	TH30360	TH30361	TH30362	TH30364	TH30366	TH30365	TH30406	TH30404
